# Association between aortic calcification and the presence of kidney stones: calcium oxalate calculi in focus

**DOI:** 10.1007/s11255-021-03058-4

**Published:** 2021-11-30

**Authors:** Bo Li, Yin Tang, Liang Zhou, Xi Jin, Yu Liu, Hong Li, Yan Huang, Kunjie Wang

**Affiliations:** 1 Department of Urology, Institute of Urology, Laboratory of Reconstructive Urology, West China Hospital, Sichuan University, Chengdu, China; 2 Health Management Center, West China Hospital, Sichuan University, Guoxuexiang 37, Chengdu, 610041 China

**Keywords:** Nephrolithiasis, Aortic calcification, Calcium oxalate calculi, Screened population

## Abstract

**Purpose:**

The current research is aimed at analyzing the relationship between kidney stone (KS) and abdominal aortic calcification (AAC) and the relationship between KS components and AAC.

**Methods:**

This is a retrospective, case–control study. Kidney stone formers (KSFs) were treated at the Department of Urology, West China Hospital, Sichuan University for urological calculus disease from January 2014 to January 2020. Matched non-stone formers (non-SFs) were drawn from the same hospital for routine health examination from January 2018 to February 2019. Research-related information was collected and reviewed retrospectively from the hospital’s computerized records. AAC were evaluated using available results of computed tomography imaging and abdominal vascular ultrasound. The relationships of AAC between KSFs and non-SFs were compared. The composition of renal calculi was analyzed by Fourier-transform infrared spectrophotometer. KSFs were divided into AAC groups and non-AAC based on AAC. The relationship of the composition of renal calculi between AAC and non-AAC were compared. The independent-sample *t* test, the chi-squared test and binary logistics regression were performed.

**Results:**

Altogether, 4516 people were included, with 1027 KSFs and 3489 non-SFs. There were no significant differences in the laboratory parameters between KSFs and non-SFs. The association between the presence of AAC and KS was significant in multivariable model 2 [adjusting hypertension, diabetes mellitus, fasting blood glucose, uric acid, serum triglyceride (TG), serum calcium, and urine pH] (OR 5.756, 95% CI 4.616–7.177, *p* < 0.001). The result of KSFs showed that calcium oxalate calculi (CaOx) was significantly associated with AAC in multivariable model 3 (adjusting age, hypertension, diabetes mellitus, drinking history, smoking history, and TG) (OR 1.351, 95% CI 1.002–1.822, *p* = 0.048).

**Conclusions:**

The current study pioneered the revelation of the relationship between CaOx and AAC. Through an elimination of the confounding factors, the study demonstrated that KS and AAC were connected.

## Introduction

Nephrolithiasis, with a high recurrence rate of more than 50% within 10 years [1], is a problem faced by many, with a prevalence of about 10% in male and 6% in female [2]. The diagnosis of kidney stones is generally relatively simple, according to the patient’s symptoms, physical examination and auxiliary examinations can be clear. Abdominal CT has become an increasingly popular choice in the diagnosis of urinary calculi, as well as providing other imaging data. Patients with urolithiasis diagnosed through non-contrast CT imaging has more than tripled in the emergency medical unit of the United States, rising to 71% [3]. The American Urological Association’s (AUA) and the European Urological Association’s (EUA) guidelines on urolithiasis strongly recommend CT imaging before surgical intervention for nephrolithiasis [4, 5].

At the same time, abdominal CT is an ideal tool to evaluate abdominal aortic calcification (AAC). In recent years, epidemiologic study have provided evidence for an association between nephrolithiasis and cardiovascular disease [6]. When using CT colonography to assess the condition of the colon, the condition of AAC is introduced to assess the risk of any coronary arterial disease [7]. The increased incidence of cardiovascular disease in kidney stone formers (KSFs) is of great interest, although the underlying mechanisms are not well understood and are mainly being explored.

Both KS and AAC are easy to evaluate. In the case of kidney stone evaluation, performing abdominal CT imaging is an efficient technique. Nonetheless, no studies have been done on the correlation between AAC and KS. The study was based on a hypothesis that there is a relationship between KS and AAC. In specific, AAC leads to a higher probability of KS occurrence, and the two may have common pathogenesis.

We retrospectively collected the conditional information of abdominal aorta in patients with KSFs and non-SFs, and the composition information of stones in patients with renal calculi. The purpose of the present research is to analyze the relationship between nephrolithiasis and AAC and the relationship between KS components and AAC, followed by a discussion on whether AAC can predict the occurrence of stones.

## Methods

### Participants

#### Kidney stone formers and non-stone formers

This is a retrospective, case–control study. KSFs were treated at the Department of Urology, West China Hospital, Sichuan University for urological calculus disease from January 2014 to January 2020. Matched non-SFs were drawn from the same hospital for routine health examination from January 2018 to February 2019. Kidney stones were diagnosed by professional physician with (non-contrast or contrast) abdominal CT, kidney–ureter–bladder X-ray photography (KUB), or urinary ultrasound.

**Fig. 1:**
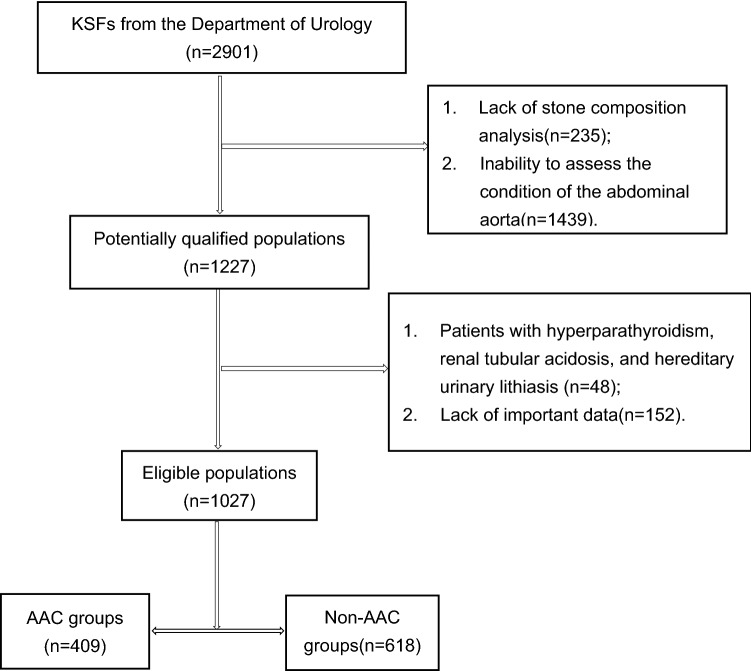
Flowchart of inclusion and exclusion of populations with kidney stones

**Fig. 2:**
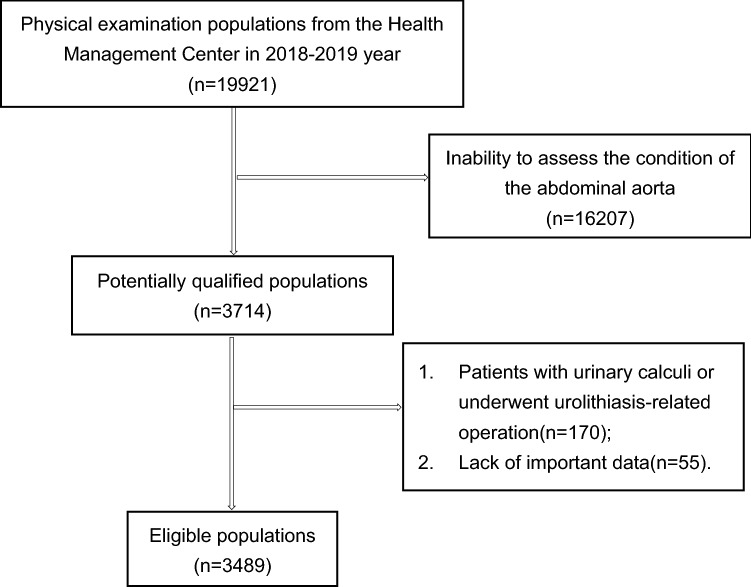
Flowchart of inclusion and exclusion of healthy physical examination populations

Demographic and clinical variables of both KSFs and non-SFs include general items (height, weight), laboratory examination (complete blood cell, basic chemistry, blood coagulation, thyroid function, stool/urine analyses), abdominal CT, abdominal aortal ultrasonography, kidney–ureter–bladder X-ray photography (KUB), or urinary ultrasound, etc. KSFs undergone stone composition analysis were included. Non-SFs were unable to evaluate aortic calcification were excluded, and were diagnosed with urinary calculi or underwent urolithiasis-related operations were also excluded. Patients with hyperparathyroidism, renal tubular acidosis, and hereditary urinary lithiasis were excluded. The screening process of patients with KSFs and non-SFs is presented in Figs. 1 and 2. The relationships of AAC between KSFs and non-SFs were compared.

#### AAC group and non-AAC group

KSFs were divided into AAC group and non-AAC group based on AAC, shown in Fig. 1. The relationship of the composition of renal calculi between AAC and non-AAC were compared.

### Data collection

Research-related information was collected and reviewed retrospectively from the hospital’s computerized records. Specifically, the following parameters were collected: age, gender, body mass index (BMI), diabetes mellitus, hypertension, smoking history, drinking history, stone size, stone location, and laboratory data: FBG, UA, TG, TC, Ca, and pH.

### Evaluation of the composition of renal calculi

The composition of renal calculi was analyzed by Fourier-transform infrared spectrophotometer (Lambda-LIIR.20, Tianjin, China), which were categorized according to EAU guidelines [8]. Evaluated by the main component, stones were divided into CaOx and carbapatite calculi (CaP). Meanwhile, a stone was treated as a urate stone (UA) if it contained uric acid, sodium urate monohydrate, or ammonium acid urate. Struvite is placed in the group of the same name, “struvite”. Cystine (*n* = 6) and drug-induced calculi (*n* = 1) were categorized as “other”.

### Measurement of abdominal aorta calcification

AAC were evaluated using available results of abdominal CT imaging and abdominal vascular ultrasound. Ultrasonographic examination of the abdominal aorta is just a supplement. To increase the accuracy of diagnosis of vascular calcification, abdominal vascular ultrasound and abdominal CT were used for mutual verification of AAC. The measurement range of the abdominal aorta starts at 1 cm from the starting point of the abdominal aorta and ends at 1 cm under iliac bifurcation. AAC exists if the aortic wall area is larger than 1 mm^2, and the density is over 130 Hounsfield unit (HU) using available CT imaging [9]. In an ultrasonographic examination of the abdominal aorta, patients needed to keep the supine position and fast before the examination. The ultrasonic probe was placed at the beginning of the abdominal aorta under the xiphoid process. The longitudinal and transverse sections of the abdominal aorta were observed from the starting point of the abdominal aorta to the iliac bifurcation. Then, the abdominal aorta was magnified routinely during the examination.

### Statistical analysis

In this study, continuous variables were calculated by means ± standard deviations (SD), whereas categorical variables were measured by counts (percentages). If the continuous variable conforms to the normal distribution, an independent-sample *t* test was performed. Otherwise, the rank sum test is used. For categorical variables, when *T* ≥ 5 and sample size *n* ≥ 40, the chi-square test was selected for data analysis. If 1 ≤ T < 5 and *n* ≥ 40, the continuity corrected chi-square test was carried out.

Binary logistics regression was utilized to assess the correlation between KS and AAC. Beyond that, multivariable model 1 was set up to adjust age and gender, and multivariable model 2 was designed to adjust age, gender, BMI, diabetes mellitus, hypertension, smoking history, drinking history, FBG, UA, TG, Ca, and urine pH, which studies have confirmed that these factors may be closely related to the occurrence and development of KS [10].

KS were divided into two groups based on AAC, to evaluate the relationship between AAC and the stone composition. Here, binary logistics regression was performed and multivariable model 3 was framed to adjust age, diabetes mellitus, hypertension, smoking history, drinking history, and TG, which these factors have been shown to be closely related to vascular calcification [11].

This was a two-tailed test and when *p* value was less than 0.05, the analysis was considered statistically significant. All statistical analyses were performed with SPSS software (SPSS Inc, Version 22, Chicago, IL, USA). The study protocol was approved by the Biomedical Ethics Committee at West China Hospital, Sichuan University (No. 2015-202).

## Results

In total, 4516 samples were included, including 1027 patients diagnosed with nephrolithiasis, and 3489 non-SFs (i.e., healthy people) as controls. Variables of interest were categorized by the presence or absence of KS, as shown in Table 1. The prevalence of AAC was higher among KSFs in comparison to non-SFs (i.e., 39.8% vs 20.2%, *p* < 0.001). Table 2 shows the results of the regression analyses set to assess the association between the presence of AAC and KS in models. There was a significant association between AAC and KSFs, (OR 2.618, 95% CI 2.254–3.041, *p* < 0.001), which was not found among non-SFs with the unadjusted model 1. In multivariable model 1 that was designed to adjust age and gender, it was observed that KSFs had higher AAC (OR 4.237, 95% CI 3.529–5.087, *p* < 0.001). In a similar vein, the relationship was also significant in multivariable model 2 which also adjusted hypertension, diabetes mellitus, drinking history, smoking history, FBG, UA, TG, Ca, and urine pH variables (OR 5.756, 95% CI 4.616–7.177, *p* < 0.001).

**Table 1:** General characteristics of the study, categorized according to kidney stone status

	All (*n* = 4516)	KSFs (*n* = 1027)	non-SFs (*n* = 3489)	*P* value
Age, year	49.0 ± 11	48.0 ± 12.8	49.2 ± 10.4	0.006
Male (%)	2749 (60.9%)	676 (65.8%)	2073 (59.4%)	< 0.001
BMI kg/m^2^ ^a^	24.2 ± 3.3	24.0 ± 3.5	24.2 ± 3.2	0.235
Hypertension (%)	945 (20.9%)	156 (15.2%)	789 (22.6%)	< 0.001
Diabetes mellitus (%)	332 (7.4%)	71 (6.9%)	261 (7.5%)	0.54
Drinking history (%)	1908 (42.2%)	214 (20.8%)	1694 (48.6%)	< 0.001
Smoking history (%)	1428 (31.6%)	289 (28.1%)	1139 (32.6%)	0.006
FBG mmol/L	5.4 ± 1.5	5.3 ± 1.2	5.4 ± 1.6	0.024
UA μmol/L	354 ± 93	363 ± 96	351 ± 91	< 0.001
TG mmol/L	1.73 ± 1.34	1.68 ± 1.20	1.74 ± 1.38	0.201
TC mmol/L	4.90 ± 0.97	4.51 ± 0.91	5.01 ± 0.96	< 0.001
Ca mmol/L^b^	2.32 ± 0.10	2.29 ± 0.12	2.32 ± 0.09	< 0.001
Urine PH	6.01 ± 0.65	6.24 ± 0.65	5.95 ± 0.63	< 0.001
AAC (%)	1113 (24.6%)	409 (39.8%)	704 (20.2%)	< 0.001

Continuous variables were calculated by means ± standard deviations and categorical variables were measured by counts (percentages)

*KSFs* kidney stone formers, *non-SFs* non-stone formers, *BMI* body mass index, *FBG* fasting blood glucose, *UA* uric acid, *TG* serum triglyceride, *TC* serum total cholesterol, *Ca* serum calcium, *AAC* abdominal vascular calcifications

^a^BMI was available in 4304 studied populations

^b^Ca was available in 4304 studied populations

**Table 2:** The results of regression analysis models assessing AAC between KSFs and non-SFs

	Unadjusted model 1	Multivariable model 1	Multivariable model 2
OR (95%CI)	2.618 (2.254,3.041)	4.237 (3.529,5.087)	5.756 (4.616,7.177)
*P* value	< 0.001	< 0.001	< 0.001

Multivariable model 1 adjusting age and gender. Multivariable model 2 adjusting for nine more parameters, namely hypertension, diabetes mellitus, drinking history, smoking history, FBG, UA, TG, Ca, and urine pH

*CI* confidence interval, *OR* odds ratio

The stone composition of 1027 KSFs was investigated, which include 705 CaOx, 222 CaP, 55UA, 37 struvite, and 8 others (i.e., 7 cystine and 1 drug-induced calculi). The baseline characteristics of KSFs based on a composition analysis are presented in Table 3. Table 4 illustrates the results of the regression analyses model which assessed the stone composition between AAC and non-AAC populations. In unadjusted analysis 2, three compositions, namely CaOx, CaP and Struvite were significantly associated with AAC (OR 1.476, 95% CI 1.121–1.944, *p* = 0.006; OR 0.667, 95% CI 0.487–0.912, *p* = 0.011; OR 0.341, 95% CI 0.148–0.785, *p* = 0.011, respectively), except for UA and others (OR 1.609, 95% CI 0.934–2.772, *p* = 0.087; OR 0.214, 95% CI 0.026–1.745, *p* = 0.15, respectively). However, only CaOx had a significant association with AAC (OR 1.351, 95% CI 1.002–1.822, *p* = 0.048), after adjustment for age, hypertension, diabetes mellitus, drinking history, smoking history, and TG.

**Table 3:** The baseline characteristics of KSFs with composition analysis

	All (*n* = 1027)	AAC (*n* = 409)	non-AAC (*n* = 618)	*P* value
Age > 30 year (%)	921 (89.7)	407 (99.5)	514 (83.2)	< 0.001
Male (%)	676 (65.8)	284 (69.4)	392 (63.4)	0.051
BMI > 28 (%)^a^	99 (12.1)	45 (13.4)	54 (11.2)	0.331
Hypertension (%)	156 (15.2)	110 (26.9)	46 (7.4)	< 0.001
Diabetes mellitus (%)	71 (6.9)	54 (13.2)	17 (2.8)	< 0.001
Drinking history (%)	214 (20.8)	106 (25.9)	108 (17.5)	< 0.001
Smoking history (%)	289 (28.1)	142 (34.7)	147 (23.8)	< 0.001
TG > 2.3 mmol/L (%)	185 (18.0)	92 (22.5)	93 (15.0)	0.002
TC > 6.2 mmol/L (%)	36 (3.5)	19 (4.6)	17 (2.8)	0.106
Size, mm	17.0 ± 8.0	17.3 ± 8.6	16.7 ± 7.5	0.233
Right kidney (%)	507 (49.4)	189 (46.2)	318 (51.5)	0.100
CaOx (%)	705 (68.6)	301 (73.6)	404 (65.4)	0.005
CaP (%)	222 (21.6)	72 (17.6)	150 (24.3)	0.011
UA (%)	55 (5.4)	28 (6.8)	27 (4.4)	0.084
Struvite (%)	37 (3.6)	7 (1.7)	30 (4.9)	0.008
Others (%)	8 (0.8)	1 (0.2)	7 (1.1)	0.155

Populations with kidney stones are divided into two groups based on the presence and absence of AAC to evaluate the relationship between AAC and stone composition

Continuous variables were calculated by means ± standard deviations and categorical variables were measured by counts (percentages)

*AAC* abdominal vascular calcifications, *CaOx* calcium oxalate calculi, *CaP* carbapatite, *UA* urate stone, *BMI* body mass index, *TG* serum triglyceride, *TC* serum total cholesterol

^a^BMI was available in 818 studied populations

**Table 4:** The results of regression analysis models assessing stone composition between AAC and non-AAC

	CaOx	CaP	UA	Struvite	Others
Unadjusted model 2					
OR (95%CI)	1.476 (1.121,1.944)	0.667 (0.487,0.912)	1.609 (0.934,2.772)	0.341 (0.148,0.785)	0.214 (0.026,1.745)
*P* value	0.006	0.011	0.087	0.011	0.15
Multivariable model 3					
OR (95%CI)	1.351 (1.002,1.822)	0.732 (0.522,1.025)	1.477 (0.809,2.696)	0.447 (0.186,1.076)	0.180 (0.017,1.933)
*P* value	0.048	0.07	0.204	0.072	0.157

Multivariable model 3 adjusting age, hypertension, diabetes mellitus, drinking history, smoking history, and TG

*CaOx* calcium oxalate calculi, *CaP* carbapatite, *UA* urate stone, *CI* confidence interval, *OR* odds ratio

## Discussion

Several interesting findings were disclosed in our research. First, multivariate analysis 2 confirmed that kidney stone disease is independently associated with AAC through a comparison of KSFs and non-SFs, despite the fact that potential confounders were noticed. Shavit L. et al. reported that KSFs with relapsing nephrolithiasis had a more severe AAC score than non-SFs (*p* < 0.001) [12]. Patients with intermediate or severe AAC was 1.9 times more likely to have KS than those without AAC (OR 1.9, *p* = 0.004) [13]. The multivariable logistic regression suggests that the formation of recurrent KS was correlated with moderate or severe coronary artery calcification (CAC) rather than none or mild CAC (OR 1.80, 95% CI 1.22–2.67) [14].

In what follows, our study is the first of its type to reveal the relationship between calcium oxalate stone and AAC after adjusting the vascular calcifications related confounding factors. Then, after controlling the KS-related confounding factors, our study still found a positive correlation between KS and AAC.

A further exploration also showed that the prevalence of KS in patients with AAC was significantly higher than non-SFs (39.8% vs 20.2%, *p* < 0.001). Karen L. Stern et al. claimed that AAC was more widespread among the KSFs [13]. Insofar, increasingly more medical literature referred to the concept of kidney calculi as a systemic disease. In a similar vein, many studies have demonstrated an association between kidney calculi and metabolic syndrome, hypertension, diabetes mellitus, or urine pH, UA [15, 16]. In fact, we found a higher prevalence of diabetes mellitus, hypertension, smoking history, and drinking history among non-SFs, whereas the prevalence of FBG, UA, or TG was similar among KSFs and non-SFs.

We discovered a higher prevalence of obesity, hypertension, diabetes mellitus, drinking history, smoking history, hyperlipidemia and hypercholesterolemia in patients with AAC, if compared with those without AAC. In the unadjusted model 2, we analyzed the relationship between the components of KS and AAC, and found that patients with AAC was 1.48 times more likely to have CaOx-composed stones than those without AAC (OR = 1.476, *p* = 0.006), whereas patients with AAC were less likely to have CaP- and struvite-composed stones than those without AAC (OR = 0.667 *p* = 0.011; OR = 0.341 *p* = 0.011). Multivariate analyses model 3 confirmed that AAC is independently associated with CaOx (OR = 1.351, 95%CI 1.002–1.822). Pietro Manuel Ferraro et al. have detected an association between AAC and CaP [17]. In this study, they used an alternative model that suggests KS components are comparable, just as protein, sugar, and fat can be easily compared based on energy calculation. However, this is not in conformity with the normal situation. Stone composition is not like protein, fat or sugar. KS components are usually a mixture. In most cases, the prominent composition of a urinary stone is CaOx, accounting for about 50–80% of the total, followed by CaP and UA [18–20]. Patel et al. revealed that calcium phosphate is associated with AAC (*p* = 0.016), however, they did not probe the correlation between the two parameters [8].

Taguchi et al. disclosed that the production of M1 macrophages was induced by an oxalate-inducing diet [21]. Liu et al. reported that the formation of atherosclerosis and insulin-resistance were accompanied by the appearance and increase of M1 macrophage [22]. In Ketha et al. study, a blood electrolyte test of patients with urolithiasis has illustrated that serum calcium and phosphate ion concentration were higher in SFs [23]. Meanwhile, the increased calcium ion concentration activates the runt-related transcription factor, thereby transforming smooth muscle cells into osteoblast-like cells; then, these osteoblast-like cells facilitates the formation of the bone morphogenic proteins and osteopontin, thus leading to vascular calcification to some extent [24]. The expression of osteopontin and monocyte chemoattractant protein-1 appeared in different types of cells with the increase of both free oxalate ions and CaOx crystal [25, 26]. Kleinman et al. reported that osteopontin was involved in the defense of endogeny against crystal formation, which was detected in both SFs and animals with urolith [27]. Constantly elevated osteopontin accelerates plaque progression, which may play a part in prognostic biomarker [28]. Osteopontin promotes atherosclerosis and inhibits vascular calcification; osteopontin insufficiency in animal models attenuates atherosclerosis [29]. Oxalate itself induces the reactive oxygen species that promote inflammation and further conduce to systemic oxidation and vascular endothelial cells injury [29, 30]. Moreover, recent research suggests that in ossification mechanism, there is a significant relationship between the active participation of proteins (i.e., osteopontin, bone sialoprotein, and bone morphogenetic protein 2) and transcription factors (i.e., core-binding factor α and runt-related transcription factor 2), which is also involved in the early phases of kidney stone formation and arterial calcification [31–35].

Furthermore, a new mechanism for the formation of kidney—mineral nanoparticles has been found in calcified blood vessels and kidney [36, 37]. Wong et al. have implied that precursors of calcification and renal stones may represent the nanoparticles in humans [36]. Both KS and aortic calcification were regarded as ectopic calcification, whose occurrence and progress may have a common underlying mechanism [38–40]. Letavernier et al. revealed that pyrophosphate deficiency might contribute to the development of vascular calcifications in KS formers [44]. The formation of Randalls plaques of calcium phosphate was taking place in basement membranes and the interstitium [41]. This course is analogous to bone formation as well as the process of coronary artery calcification [42].

Some limitations must be taken into account when interpreting the findings of the study. The composition of stones cannot be quantified, so the study could only reveal the main components of stones. Beyond that, since this is a retrospective study, data collection might have been influenced by some unknown confounding factors. For instance, a participant diagnosed with KS may have changed the previous unhealthy lifestyle and exercised more, which is likely to change the profile of confounders that influence the occurrence and development of AAC. It is also true that the accurate time of AAC and KS formation could not be evaluated, as this is a cross-sectional study. Hence, it is necessary to carry out longitudinal studies to understand this relationship in a more comprehensive manner. Moreover, patients with chronic diseases like hypertension and diabetes usually take drugs for a long time, which might also affect the formation kidney stones and AAC.

In conclusion, this study blazed the trail to investigate the correlation between calcium oxalate stone and AAC after an adjustment of confounding factors. The analyses show that KS and AAC are closely associated. Patients with AAC are more likely to have kidney stones. However, whether aortic calcification can predict KS needs further exploration. In a similar vein, the specific pathophysiological mechanisms of KS and aortic calcification need to be further clarified.

## Data Availability

All original research materials are kept by corresponding author.
